# Effect of HTST and Holder Pasteurization on the Concentration of Immunoglobulins, Growth Factors, and Hormones in Donor Human Milk

**DOI:** 10.3389/fimmu.2018.02222

**Published:** 2018-09-27

**Authors:** Diana Escuder-Vieco, Irene Espinosa-Martos, Juan M. Rodríguez, Leónides Fernández, Carmen Rosa Pallás-Alonso

**Affiliations:** ^1^Banco Regional de Leche Materna, Hospital Universitario 12 de Octubre, Instituto de Investigación i+12, Madrid, Spain; ^2^Probisearch S.L., Tres Cantos, Spain; ^3^Sección Departamental de Nutrición y Ciencia de los Alimentos (Veterinaria), Universidad Complutense de Madrid, Madrid, Spain; ^4^Sección Departamental de Farmacia Galénica y Tecnología Alimentaria (Veterinaria), Universidad Complutense de Madrid, Madrid, Spain; ^5^Servicio de Neonatología, Hospital Universitario 12 de Octubre, Instituto de Investigación i+12, Universidad Complutense de Madrid, Madrid, Spain

**Keywords:** donor milk, HTST pasteurization, Holder pasteurization, immunoglobulins, growth factors, hormones

## Abstract

Donor human milk (DHM) is submitted to Holder pasteurization (HoP) to ensure its microbiological safety in human milk banks but this treatment affects some of its bioactive compounds. The objective of this work was to compare the effects of HoP and high temperature short time (HTST) treatments on some bioactive compounds found in DHM. A total of 24 DHM batches were processed in a continuous HTST system (70, 72, and 75°C for 5–25 s) and by HoP (62.5°C for 30 min). The concentrations of immunoglobulins (Igs) A, G, and M, transforming growth factor-beta 2 (TGF-β_2_), adiponectine, ghrelin, and leptin were measured using a multiplex system, whereas the concentration of epidermal growth factor (EGF) was determined by ELISA. In relation to Igs, IgG showed the highest preservation rates (87–101%) after HTST treatments, followed by IgA (54–88%) and IgM (25–73%). Ig retention after any of the HTST treatments was higher than after HoP (*p* < 0.001). Treatment times required to reduce the concentration of IgM by 90% (*D*-value) were 130, 88, and 49 s at 70, 72, and 75°C, while the number of degrees Celsius required to change the *D*-value by one factor of 10 (*z*-value) was 11.79°C. None of the heat treatments had a significant effect on the concentrations of TGF-β_2_, EGF, adiponectin, and ghrelin. In contrast, leptin was detected only in 4 of the samples submitted to HoP, whereas it was present in all samples after the different HTST treatments, with retention rates ranging between 34 and 68%. Globally, the concentration of IgA, IgG, IgM, and leptin in DHM was significantly higher after HTST pasteurization performed in a continuous system designed to be used in human milk banks than after the HoP procedure that is routinely applied at present.

## Introduction

Mother's own milk (MOM) is the gold standard for infant feeding in early life. It provides the macronutrients and micronutrients that fulfill the nutritional requirements of the newborn and, also, a myriad of bioactive compounds, such as immunoglobulins (Igs), cytokines, growth factors (GFs), and hormones (1–4). In fact, human colostrum and milk are often regarded as the most important sources of natural bioactive compounds, which are associated to a wide variety of physiological functions in the breastfeeding infant. Such functions include protection against infections, establishment of an efficient gut barrier and training of the infant immune system, favoring the development of intestinal and systemic homeostasis (5). The delivery of immune compounds and GFs into the rapidly developing infant gut may prevent many adverse outcomes, including necrotizing enterocolitis (NEC) and long-term cardiovascular risk, particularly in preterm or very low birth weight infants (6, 7). Moreover, the protection that breastfeeding confers against faster weight gain and, consequently, against later obesity appears to be associated to the presence in milk of hormones involved in food intake regulation and energy balance (8). All these bioactive compounds present in human milk are absent in infant formulas. Therefore, when MOM is unavailable, or is in short supply to meet the nutritional requirements of the preterm infant, the next best alternative is donor human milk (DHM) (9, 10).

DHM is usually pasteurized to ensure its microbiological safety and, currently, Holder pasteurization (HoP) is the most common method being applied in human milk banks (HMB). This method, which involves heating DHM at 62.5°C for at least 30 min, destroys high-risk viruses and non-spore-forming bacteria (11). Unfortunately, some bioactive compounds present in human milk are affected by HoP to varying degrees. In particular, HoP reduces the concentrations and biological activities of all Ig classes, probably due to their complex and delicate structure, which is crucial to carry out their specific biological functions (12). Among Igs, IgA is the most abundant in human milk and, therefore, has been the most extensively studied regarding the effect of heat treatment on its concentration, which is reduced by 20–60% after HoP (12–16). Other Ig classes, such as IgGs and IgM, have not been examined in detail although their levels also seem to decrease after HoP (12). On the other hand, some GFs, such as epidermal growth factor (EGF) and transforming growth factor-beta (TGF-β), can withstand the conditions of HoP. However, the low number of studies and the remarkable variability in structure between the different GFs do not allow to draw a clear conclusion about their sensitivity to heat treatments (12, 17–21). Furthermore, although the physiological effects of hormones depend mostly on their concentration, few studies have evaluated the variation in the hormone content of human milk after pasteurization. It has been reported that adiponectin concentration in human milk decreases after HoP while that of leptin does not change (12) but, again, the scarce information available does not allow to draw any conclusion.

Alternative methods for DHM treatment are been investigated in order to ensure a proper microbial inactivation, while improving the preservation of its bioactive components (22). Among them, high temperature short time (HTST) pasteurization seems the one with a higher retention of IgA, bile salt-stimulated lipase, lactoferrin, and lysozyme in DHM (23–25). Nevertheless, previous studies involved a low number of samples or the use of experimental systems that may be unsuitable for routine use in HMBs. In this context, a continuous HTST system to pasteurize DHM in the HMB operating environment was designed and validated recently (26). This HTST equipment was conceived to ensure accurate, simple and flexible operation at a HMB setting where the available amount of DHM may be variable. In addition, processing of DHM at 72°C for, at least, 10 s in this HTST system allowed to achieve the microbiological safety objectives currently established in HMBs (26).

Therefore, the objective of this work was to compare the effects exerted by the routine HoP treatment or HTST treatments performed in the cited system on the concentration of some of the main Igs, GFs and hormones present in donor milk. In addition, the effect of HTST pasteurization at different temperature/time pairs on these bioactive compounds was also evaluated.

## Materials and methods

### Human milk samples

DHM samples were obtained from the Regional Human Milk Bank “Aladina-MGU” located at the Hospital Universitario 12 de Octubre (Madrid, Spain). Milk collection was performed following a specific protocol for donor mothers approved by the Hospital 12 de Octubre Clinical Research Ethics Committee (ethical approval code: 12/325); informed consent was obtained from each donor in accordance with the Declaration of Helsinki. Milk was collected at home, frozen (−18°C) in a domestic refrigerator, and transported to the HMB in an insulated box provided with ice packs.

### Experimental design

Milk samples collected from multiple donors were thawed in a shaking water bath (at 37°C) and mixed to compose a production batch (10 L; around 12 donors per batch). A total of 24 DHM production batches were used in this study. An aliquot (120 mL) of pooled raw milk from each production batch was used as control (raw milk). Another aliquot (120 mL) was transferred to a glass bottle (150 mL), and subjected to HoP (62.5°C for 30 min) and fast cooling at 4°C in shaking water baths (Jeio Tech BS-21, Lab Companion, Oxfordshire, UK) following the current HMB protocol. The rest of the production batch was HTST-processed at a fixed temperature (70, 72, or 75°C) and different times (5, 10, 15, 20, and 25 s) using the HTST equipment described by Escuder-Vieco et al. (26). A total of 8 batches were HTST-processed at 70°C, 9 at 72°C, and 7 at 75°C.

All aliquots of untreated (raw) or heat-processed (both HoP and HTST) DHM were stored frozen (−20°C) until analysis. To avoid interferences in immunoassays, the fatty layer was removed from samples by centrifugation at 14,000 × g for 10 min at 4°C.

### Analysis of Igs, GFs, and hormones in the milk samples

Concentrations of Igs (IgA, IgG, and IgM), transforming growth factor-beta 2 (TGF-β_2_) and hormones (adiponectin, ghrelin, and leptin) were determined in duplicate using a Bioplex 200 system instrument (Bio-Rad, Hercules, CA, USA) and the Bio-Plex Pro Human Isotyping Assay, Bio-Plex Pro Human TGF-β Assay and Bio-Plex Pro Human Diabetes Assays kits (Bio-Rad). The concentration of EGF was measured using the RayBio® Human EGF ELISA kit (RayBiotech, Norcross, GA). Every assay was performed according to manufacturer's instructions. Standard curves were performed for each analyte. The analytes were assayed in, at least, three batches for each HTST treatment.

The inter-assay coefficients of variation were below manufacturers' instructions for all the immune markers, and the lower limit of quantification (LLOQ) for every analyte in human milk were: 0.21 μg/L for IgA, 0.78 μg/L for IgM, 2.19 μg/L for IgG, 1.57 ng/L for TGF-β_2_, 0.03 ng/L for EGF, 23.10 ng/L for adiponectin, 7.40 ng/L for ghrelin, and 11.45 ng/L for leptin.

### *D*- and *z*-values

*D*-value, or decimal reduction time, is the time required at any given temperature to denature 90% of a biological compound. It was estimated graphically after plotting active IgM concentrations (log_10_) against treatment times at each temperature (70, 72, or 75°C). The average slope of the resultant curves were obtained using linear regression analysis and used to calculate the *D*-value at each temperature treatment using the following equation:

$$\begin{array}{l} D=-1/slope. \end{array}$$

The variation of *D*-value with a change in temperature is indicated by the *z*-value or thermal denaturation constant. The *z*-value is the change (increase or decrease) in temperature necessary to change (reduce or increase) the *D*-value by a factor of 10. This parameter was obtained after plotting the logarithms of the *D*-values versus the corresponding temperatures at which the *D*-value was obtained. The *z*-value was the absolute value of the reciprocal of the slope of this graph.

### Statistical analysis

Normality of data distribution was tested through Saphiro–Wilks tests. Results are displayed as the median and interquartile range (IQR). The effect of heat treatments on Igs, GFs, and hormones concentration was expressed as the retention rate in relation to raw DHM samples. Kruskal–Wallis test was used to compare the effect of HTST treatments and HoP on the concentration of these bioactive compounds. Pairwise *post-hoc* multiple comparisons with the Bonferroni correction were used to identify which treatments were significantly different. The effect of temperature and time, as well as the interaction temperature × time, used for HTST treatments on the inactivation of Igs, GFs, and hormones in milk was evaluated using multifactorial analysis of variance. Linear regression analysis was performed to calculate *D*- and *z*-values.

Significance was set at *p* < 0.05. Statgraphics Centurion XVII version 17.0.16 (Statpoint Technologies, Inc., Warrenton, VA, USA) and R 3.3.2 (R-project, http://www.r-project.org) software were used to perform these analyses.

## Results

### Igs, GFs, and hormones in raw DHM

All the Igs, GFs, and hormones analyzed in this work were detected in all the batches of raw DHM and showed a wide range of concentrations (Table 1). Igs were the most abundant ones (in the mg/L range) while GFs and adiponectin were present at relatively moderate concentrations and, finally, ghrelin and leptin displayed the lowest levels (in the μg/L to ng/L range). Median concentration of IgA (473.32 mg/L) was more than 20-fold that of IgG and IgM in raw DHM batches (17.99 and 18.79 mg/L, respectively). The concentrations of EGF and TGF-β_2_ oscillated from 3.14 to 4.01 μg/L and from 446.36 to 4592.45 μg/L, respectively. Among hormones, adiponectin was more abundant (7.74 μg/L) than ghrelin and leptin (28.24 and 116.97 ng/L, respectively). IQRs of Igs, GFs, and hormones indicated a wide variation in the concentration of these compounds in the raw DHM batches. The coefficients of variation, being scale insensitive, allowed comparing the diversity in the content of these compounds in DHM. The highest and the lowest heterogeneity of the concentration values were found for IgM (85.33%) and EGF (10.27%), respectively. The coefficient of variation of other compounds was higher than 35%.

**Table 1:** Concentration of immunoglobulins, growth factors, and hormones in raw DHM batches.

	***n***	**Median**	**IQR**	**Range**	***CV* (%)**
				**Minimun–Maximun**	
**IMMUNOGLOBULINS**					
IgA (mg/L)	18	473.32	381.51–694.43	109.88–905.02	43.21
IgG (mg/L)	17	17.99	13.59–46.84	7.80–75.95	69.98
IgM (mg/L)	20	18.79	9.22–47.46	5.06–87.26	85.33
**GROWTH FACTORS**					
EGF (μg/L)	6	3.51	3.24–3.99	3.14–4.01	10.27
TGF-β_2_ (μg/L)	21	1.84	1.20–2.45	446.36–4592.45	50.76
**HORMONES**					
Adiponectin (μg/L)	24	7.74	6.41–9.27	3.74–17.6	36.68
Ghrelin (ng/L)	15	28.24	16.25–43.93	10.55–57.84	49.97
Leptin (ng/L)	20	116.97	79.05–184.50	68.4–348.19	53.14

*n, number of DHM batches analyzed; IQR, interquartile range (Q25–Q75); CV, coefficient of variation, EGF, epidermal growth factor; Ig, immunoglobulin; TGF-β_2_, transforming growth factor-beta 2*.

### Comparison of HTST treatments and HoP on bioactive compounds in DHM

**Figure 1:**
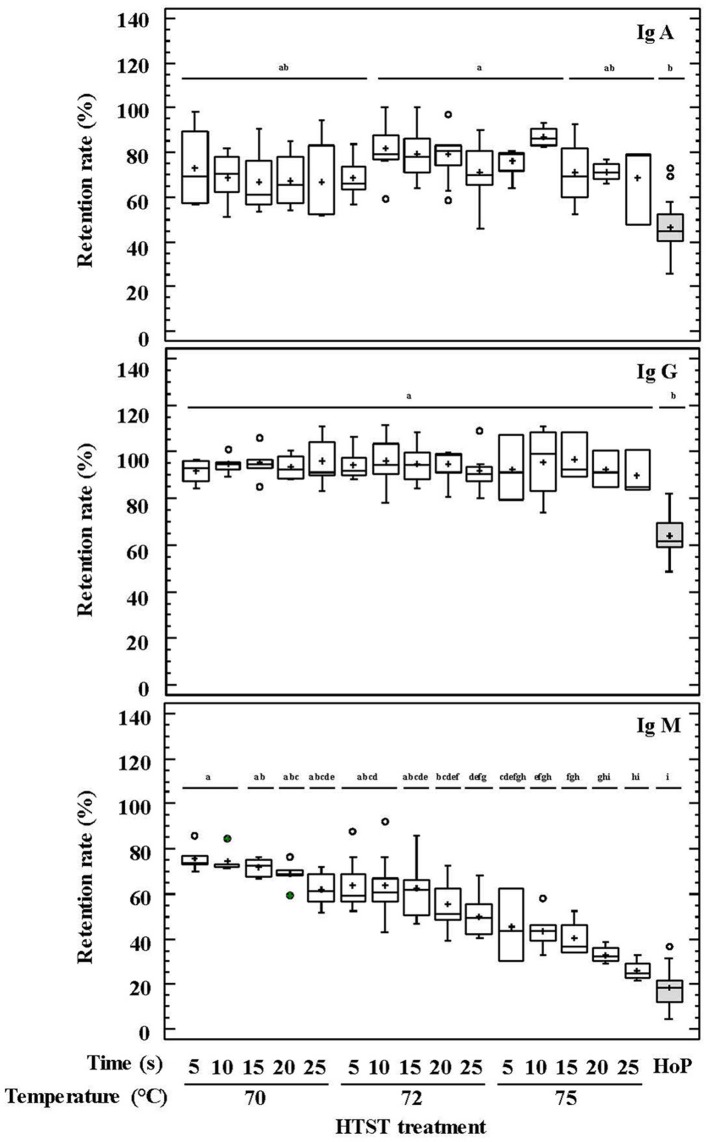
Percentage of IgA, IgG, and IgM retention in DHM before and after HTST treatment (at 70, 72, and 75°C for 5, 10, 15, 20, 25 s) or Holder pasteurization (62.5°C, 30 min). Different letters indicate a significant difference among retention rates.

The percentages of retained Ig concentrations in DHM after the different HTST treatments and HoP are shown in Figure 1. IgG showed the highest preservation rate (87–101%) after HTST treatments, followed by IgA (54–88%) and IgM (25–73%). For all Igs, the concentration values that were retained after the different HTST treatments were significantly higher than those obtained after HoP (*p* < 0.001; Figure 1).

**Figure 2:**
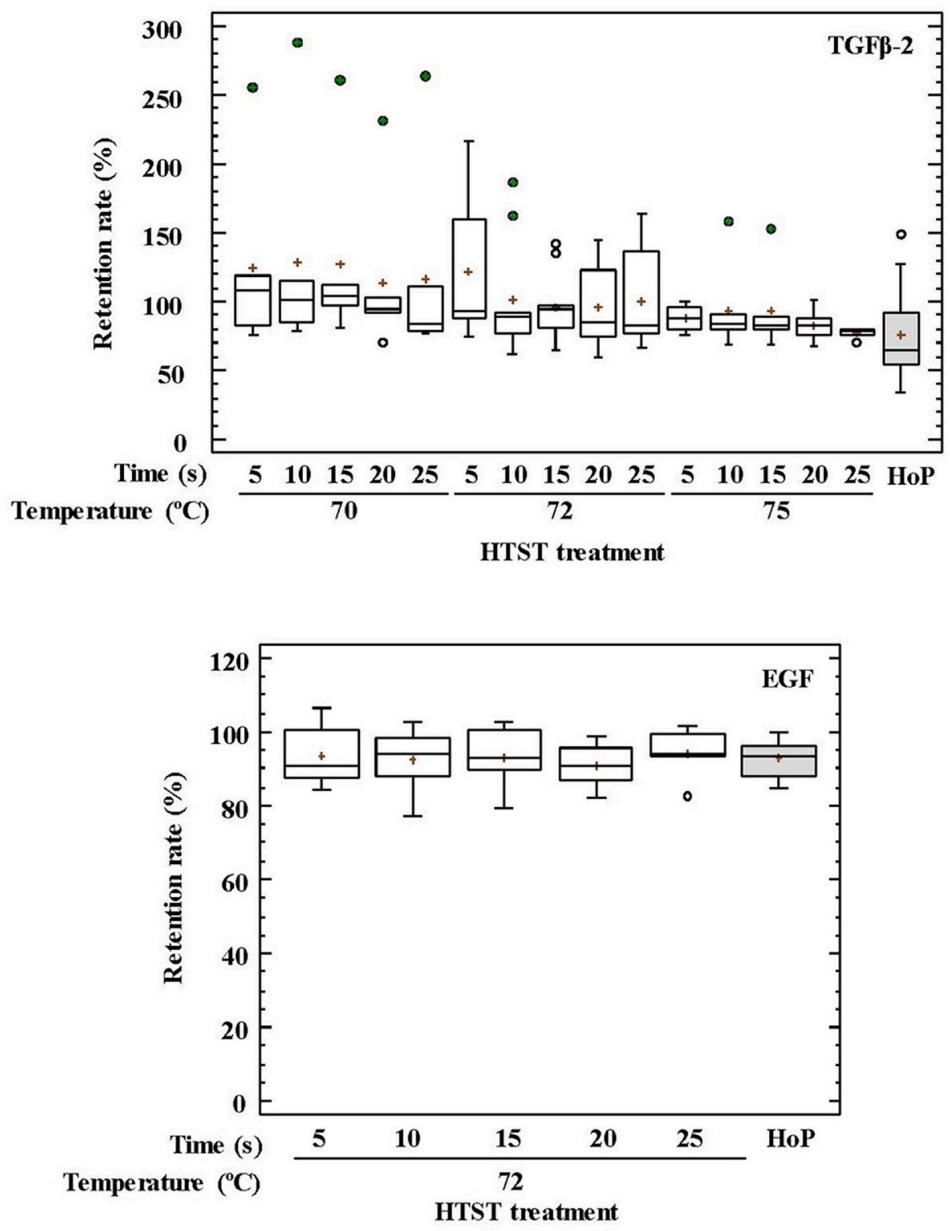
Percentage of transforming growth factor-beta 2 (TGF-β_2_) and epidermal growth factor (EFG) retention in DHM before and after HTST treatment (at 70, 72, and 75°C for 5, 10, 15, 20, 25 s) or Holder pasteurization (62.5°C, 30 min).

Regarding GFs, the retained TGF-β_2_ concentration oscillated between 78 and 107% for HTST-treated DHM and 54–92% after HoP (*p* = 0.051) (Figure 2). The impact of thermal treatments on the EGF concentration of DHM was only studied after HTST treatment at 72°C for 5–25 s or HoP, and no significant differences were found among them (*p* = 0.956) (Figure 2).

**Figure 3:**
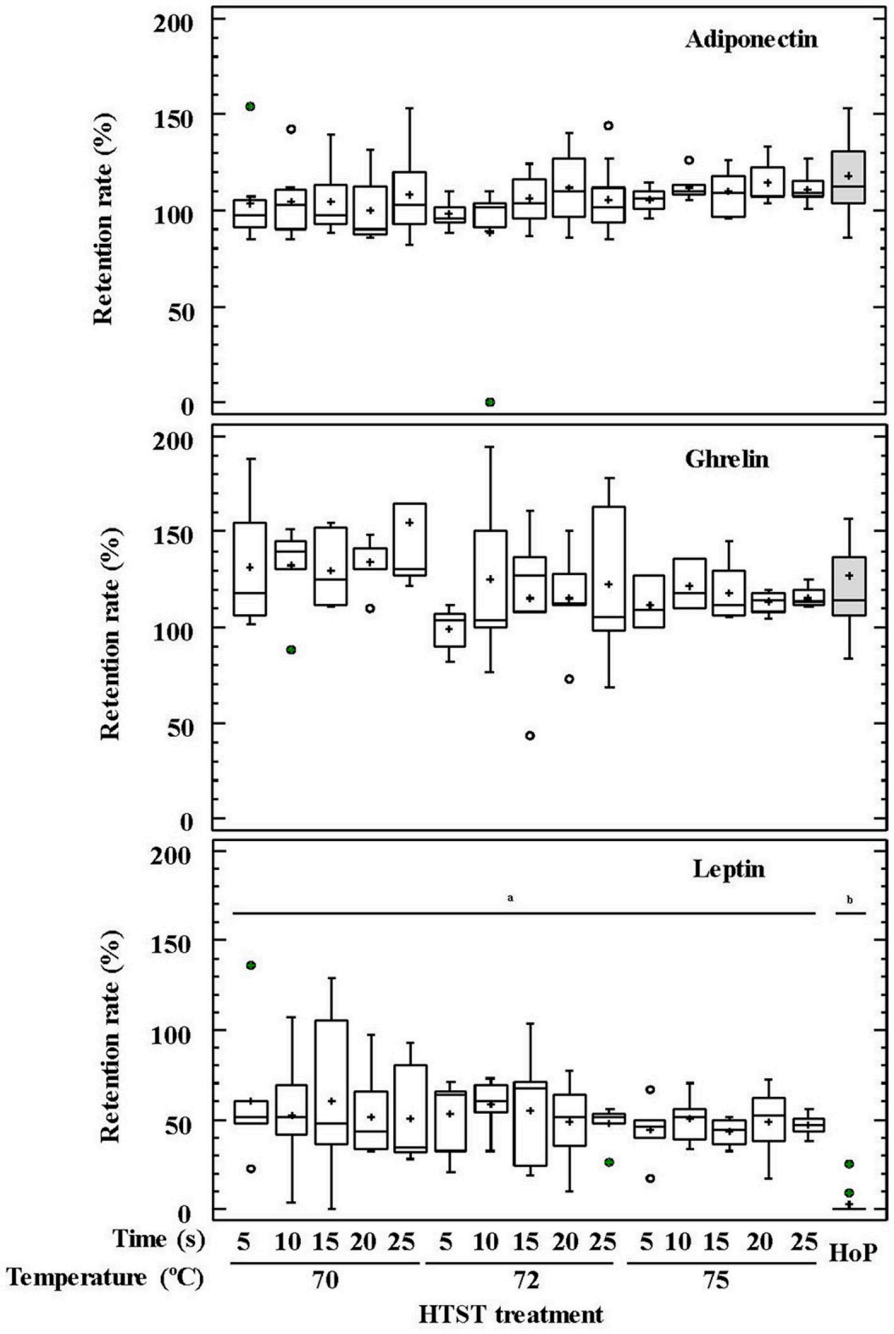
Percentage of adiponectin, ghrelin, and leptin retention in DHM before and after HTST treatment (at 70, 72, and 75°C for 5, 10, 15, 20, 25 s) or Holder pasteurization (62.5°C, 30 min). Different letters indicate a significant difference among retention rates.

No differences were observed between HoP and the HTST treatments in relation to the concentrations of adiponectin and ghrelin in DHM (*p* = 0.076 and *p* = 0.377, respectively) (Figure 3). In contrast, leptin was detected only in 4 out of 20 samples after HoP, with a mean retention rate of 13%, whereas it was present in all samples after the different HTST treatments at retention rates oscillating between 34 and 68% (Figure 3).

### Effect of the HTST processing parameters on bioactive compounds in DHM

IgA and IgG retention values showed no differences among the HTST treatments carried out at different temperatures and times, as it was confirmed by a two-way ANOVA test with temperature and time as factors (Supplementary Table 1). In contrast, IgM was less resistant to heat treatment, and its retention rate was higher when lower temperatures and times were used during the HTST treatment (two-way ANOVA: *F* = 65.46, *p* = 0.000 for temperature and *F* = 7.53, *p* = 0.000 for time), although there was no interaction between both factors (*F* = 0.15, *p* = 0.996) (Supplementary Table 1). This trend was also noted in the pairwise multiple comparison analyses performed for all heat treatments (Figure 1). *D*-values were calculated to describe the thermal destruction of IgM (Table 2), being 130, 88, and 49 s at 70, 72, and 75°C, respectively. The graphic representation of the effect of temperature on *D*-values for IgM was a perfect straight line (log_10_ D = −0.0848^*^Temperature + 8.0465; *r*^2^ = 0.999). From this graph the *z*-value for IgM was deduced to be 11.79°C.

**Table 2:** *D*-values (s) for IgM at different temperatures.

**Temperature (°C)**	***K*-value (s)**	***D*-value (s)**	***r*^2^**
70	−0.0077	130	0.842
72	−0.0113	88	0.867
75	−0.0203	49	0.821

The impact of temperature and time during HTST treatments on the retention rates of GFs and hormones was also analyzed using two-way ANOVA tests (Supplementary Table 1). Only temperature had an influence on the retention rates of TGF-β_2_ and ghrelin, and the recorded concentration for both compounds after HTST treatment was higher than for unprocessed DHM. The mean retention rates were 121% at 70°C, 103% at 72°C, and 87% at 75°C for TGF-β_2_, and 136% at 70°C, and about 115% for 72 and 75°C for ghrelin.

## Discussion

In this work, the effect of HMB-operative HTST treatment on the concentration of some milk bioactive factors, including Igs, GFs, and hormones, was assessed and compared to that exerted by the traditional HoP method. Globally, HTST treatments were associated to a significantly higher preservation of, at least, some of the biologically active compounds tested in this study. The effect of this HTST system on the microbiological quality of DHM had been evaluated previously (26).

Human milk provides a high number of bioactive factors with immunological, anti-inflammatory and anti-infectious properties (27). Some of them participates in the immune protection at the mucosal level while others are immunomodulating or growth-stimulating, contributing to the correct development of the infant's mucosal barriers and immune system (5, 28–31).Overall, such a plethora of biologically active compounds seems to be responsible for the unparalleled long-lasting protection that human milk confers to infants, especially to those born prematurely, against a wide array of diseases in both developed and developing countries (32, 33).

Secretory IgA (SIgA) is the predominant Ig class found in human milk and its role in the prevention of respiratory and gastrointestinal infectious diseases in breast-fed infants is well documented (34). In addition, SIgA plays a role in the regulation of the immune response to dietary antigens and, in fact, epidemiological studies have reported an inverse relationship between the levels of maternal milk sIgAs and the development of allergy (35, 36). It must be highlighted that both the amount and repertoire of IgAs directly produced by infants are clearly deficient because antigen-exposed memory cells have not been generated yet and transplacental transfer of IgAs is very low in the human species. This situation is aggravated in preterm neonates because their earlier exposure to foreign microorganisms and antigens does not accelerate the expression of the gene pool involved in IgA production (37).

IgMs are also relevant for neonatal health playing an important role in the opsonization of Gram-negative pathogens (28, 34). Similarly, to IgAs, IgGs produced by infants are not enough for proper homeostasis during early life. IgG transplacental transfer only partially corrects this deficiency since passively acquired IgGs decrease rapidly after birth, making infants particularly sensitive to encapsulated organisms (28). All these facts reinforce the value of human milk for neonates, and particularly for those born preterm.

The levels of Igs in human milk are highly variable depending, among other factors, on the stage of lactation, decreasing rapidly in the first 4 weeks post-partum (38). The variability of the IgA content among the DHM samples analyzed in this study (381.51–694.43 mg/L) is similar to that reported for mature milk samples obtained from single individuals (247–488 μg/mL) by other authors (39, 40). Therefore, pooling donor milk from a significant number of individuals (about 12 donors per batch in this study) may not be enough to balance the differences between single individuals in the concentration of this and other compounds analyzed in this study.

Igs are thermolabile compounds but the advantage of pasteurization at high temperatures over HoP for the preservation of IgA shown in this study has been widely acknowledged (25, 41–44). In contrast, the effect of HTST treatments on other Ig classes, such as IgG or IgM, has not been evaluated previously. This study has shown the statistically significant advantage of HTST over HoP in relation to the preservation of these two Ig classes. Previous studies indicated that IgMs and IgGs are highly and moderate sensitive, respectively, to HoP (21, 45–49). In contrast, IgG was highly resistant to any of the HTST treatment applied in this work, while inactivation of IgM depended on both processing parameters, temperature and time. The ${\text{D}}_{72^{{}^{\circ}}\text{C}}$ value for IgM was 88 s, about 2.32 times lower than that for bovine IgM (205 s), and, also, there are slight differences when comparing the *z*-values for bovine (5.17°C) and human (11.79°C) IgMs (50). These results indicate that bovine IgM is more resistant than human IgM to inactivation at 72°C, but it is more sensitive to temperature changes. However, caution should be taken when comparing the results from both studies because of different experimental conditions and the large effect of equipment and medium composition on the thermal stability of Igs (51). The experimental setting in our study did not allow calculating the kinetic parameters of thermal destruction for IgA and IgG.

A varying degree of Ig preservation was observed among the samples analyzed in this study when they were submitted to the same heat treatment. This fact may be related to the different Ig concentrations that each sample had before treatment since protein denaturation is a concentration-dependent process (52). This result is in agreement with those provided by previous studies showing very variable Ig-retained levels in DHM samples after HoP (12). Heat processing of DHM in this HTST system at 72°C for 10 s, which is the minimum treatment required to ensure the microbiological safety of DHM (26), would lead to the loss of approximately 20% of IgA, 1% of IgG, and 40% of IgM concentrations.

Breastfeeding has been shown to prevent NEC and neonatal nosocomial infections in preterm infants in neonatal intensive care units (NICUs) worldwide (53, 54). However, the preventive effect of MOM against nosocomial infections has not been observed in most studies carried out using pasteurized DHM (55). This fact has been linked to differences in bioactive compounds between MOM and DHM (56). In this context, high Igs concentrations in HTST-treated DHM could help to reduce the incidence of infectious disease outbreaks in NICUs and, therefore, decrease both short- and long-term morbidity.

Human milk is rich in GFs, including TGF-β_2_ and EGF. Presence of high concentrations of TGF-β_2_ is a common feature of human milk under physiological conditions (30). TGF-β is considered as a key immunomodulatory factor in human milk (57, 58) and its importance is highlighted by the fact that endogenous gut TGF-β synthesis is defective in the neonate (59). This factor confers protection against wheeze and atopic dermatitis in breastfed children (3, 60) and plays a critical role for oral tolerance induction and the global regulation of intestinal immune responses after food ingestion (61, 62). In addition, TGF-β_2_ specifically attenuates IL-1β-induced inflammatory responses and, through interaction with endotoxin, regulates homeostasis via IL-8 levels in the immature intestine (63, 64). EGF present in human milk has a protective effect against severe neonatal intestinal diseases, such as NEC (65), due to its well-known role in altering the balance of pro- and anti-apoptotic proteins (66). Additionally, EGF enhances proliferation and differentiation of epithelial cells in the gastrointestinal tract (67), participates in the healing of damaged mucosa after injury (68, 69), and may contribute to the increased size of thymus in breastfed infants (70).

Globally, TGF-β_2_ concentrations were higher at lower HTST temperatures and diminished as temperature and/or time of treatment increased, which is in agreement with previous findings (19). TGF-β_2_ retention rates after HoP of DHM samples were generally lower than those obtained after any HTST treatment (70, 72, and 75°C for 5–25 s), although the high variation in retention rates observed among treatments resulted in non-significant differences. This high variation and, in some cases, the increase of the relative TGF-β_2_ concentration after heat treatment were probably due to either TGF-β_2_ release from milk cells during frozen storage (19), to the partial heat activation that occurs between 1 and 10 min at 60–100°C (71) or to the release of TGF-β_2_ from chondroitin sulfate-containing proteoglycans by different known activators (72, 73). Measured TGF-β_2_ concentrations in this study may be underestimated as they were related to the molecules retained in the aqueous phase of the treated samples. Since the structure of the active TGF-β_2_ form is highly hydrophobic, an important amount of this compound is associated to the fat fraction of the milk (74).

EGF retention rates did not change significantly during heat treatments. This fact may be related to its disulfide bonding patterns that determine a high conformational stability of the tertiary structure (17, 75). This high stability ensures the resistance of this GF to the conditions that it may face during its transit through the infant gastrointestinal tract and may explain, at least partly, the preventive role of DHM against NEC (76).

The presence of hormones and GFs in human milk has been linked to the function of the mammary gland as an extra-uterine extension or replacer of the placenta to regulate the activities of various tissues and organs of the infant until the maturation and full function of his own endocrine system (77). Milk hormones involved in the regulation of energy balance and metabolic development of the neonates, such as leptin, ghrelin, and adiponectin, have received considerable attention in the context of the current metabolic syndrome and obesity epidemics (78–80). Specific receptors for such hormones have been identified in the gastrointestinal mucosa (81). Therefore, it is not strange that breastfeeding has been associated to protection against faster weight gain and, consequently, later obesity (82).

Adiponectin, having an appetite-stimulatory effect and, also, regulating energy metabolism, displays the highest concentration in human milk among appetite hormones, and its concentration is reduced over the course of lactation (83, 84). Its survival after digestion, also related to high glycosylation, and the presence of adiponectin receptors in the infant gut favor its bioavailability (83). Structure of human milk adiponectin corresponds to the 18-mer form and it is associated with the highest activity (85). The adiponectin concentration in human milk is related to serum adiponectin and, inversely, to adiposity in breastfeed infants over the first 6 months of life, suggesting a role in early regulation of neonatal infant weight gain (83).

Ghrelin is involved in the short-term regulation of feeding and in the long-term regulation of weight and energy metabolism (80). Ghrelin levels in human milk increase progressively from birth through, at least, 6 months postpartum although current evidences linking its concentration in milk and specific infant outcomes are scarce (86). This 28-amino acid hormone is *n*-octanoylated and this modification (which is essential for its activity) determines high affinity for membranes, explaining why ghrelin levels are higher in whole milk than in skimmed milk (82, 87). Therefore, it is possible that ghrelin levels detected in milk samples after the defatting centrifugation step are actually lower than those present in whole milk, as it is consumed by the infants.

Leptin, which displays an anorexigenic effect, influence both short- and long-term regulation of energy balance and food intake. This hormone can be transferred from blood to milk before being secreted by epithelial cells in milk fat globules during lactation (88, 89). In fact, leptin levels in whole milk are 2- to 66-fold higher than in skimmed samples (8). This hormone is the milk component most associated to maternal body mass index (BMI), and correlates positively with infant serum leptin; the later does with infant BMI and weight (89).

Leptin was highly affected by heat treatment and, therefore, it could not be detected in any DHM sample after HoP. Its retention percentages after HTST treatments were the lowest among the hormones studied in this work. In contrast, the concentrations of ghrelin and adiponectin remained unchanged after any of the heat treatments. The increase of these hormones after both HoP and HTST treatments may be attributable to adiponectin dissociation from trimeric to monomeric forms (85) and/or to cell membrane disruption of epithelial cells present in DHM after heat treatment, favoring their release. Conflicting results regarding the impact of DHM pasteurization on hormone concentrations have been reported, ranging from reduced levels of adiponectin and insulin (90) to no differences on leptin concentration after HoP (91). Therefore, further studies are needed to confirm the effect of processing on the concentration of these modulatory compounds as well as the biological significance of changes in appetite hormone levels during early life.

The analytical method employed in this study is based on a standard sandwich enzyme immunoassay approach, which is the most popular technique to determine the concentrations of immunological compounds in biological fluids. The multiplexing assay enables the simultaneous analysis of different compounds from a small amount of sample. However, this methodology does not discriminate between biologically active and inactive molecules carrying the antigenic determinant. Therefore, bioassays must be performed in future works to test the biological activity of the analyzed compounds after the heat treatments. These complementary bioassays are expensive and time-consuming but may help to define more precisely the role of these molecules in clinical practice. In addition, there are a high number of maternal and infant factors that influence the levels of bioactive compounds in human milk and their functions in the infant gut. These factors pinpoint the difficulty in relating unequivocally the variation of one compound to a specific infant outcome (86, 92). From a methodological point of view, care must be taken to compare some of the obtained results because of the strong association of some compounds to the milk fat fraction, which was discarded during sample preparation. Nevertheless, the results obtained in this study are promising and it would be desirable to conduct a clinical trial in order to identify the clinical benefits for preterm infants related to the administration of pasteurized DHM using the continuous HTST system (26) instead of HoP-treated DHM.

## Conclusion

A wide variation in the concentration of several bioactive compounds (IgA, IgG, IgM, EGF, TGF-β_2_, adiponectin, ghrelin, and leptin) was found in raw DHM. HoP (62.5°C, 30 min) had an important impact on the concentrations of most of the bioactive compounds studied, in particular on those of IgA, IgM, IgG, and leptin, as indicated by their low remaining levels or their lack of detection after the treatment. In contrast, HTST treatment of DHM resulted in higher preservation of these compounds. IgG was the most thermostable Ig, followed by IgA, which concentration showed a reduction of ~30% independently of the temperature-time combination applied. IgM was the most susceptible Ig to the processing conditions, although about 50% of its concentration (was preserved after heating at 72°C for 10–15 s, conditions that allow to achieve the microbiological safety objectives currently established in HMBs. Leptin was also sensitive to the thermal treatment but, again, a 50% of the initial content remained after any of the HTST treatments applied in this work. The DHM concentrations of TGF-β_2_, EGF, adiponectin, and ghrelin were not affected by any heat treatment applied in this study. As a conclusion, milk quality after HTST pasteurization using the continuous system developed by Escuder-Vieco et al. (26) seems clearly better than that processed following the standard HoP procedure since the former allowed the preservation of higher concentrations of the bioactive compounds analyzed in this work.

## Author contributions

DE-V participated in the design of the study, acquisition of the samples, carried out the pasteurization processes, performed the immunological assays, analyzed and interpreted the data, and drafted the manuscript. IE-M participated in the design of the study, carried out the analyses, and participated in the data analysis. JR designed the study and provided critical revisions of the manuscript for important intellectual content. CP-A and LF participated in the design of the study, funding acquisition, analysis of the data, and provided a critical revision of the manuscript. All authors read and approved the final manuscript.

### Conflict of interest statement

The authors declare that the research was conducted in the absence of any commercial or financial relationships that could be construed as a potential conflict of interest.

## Abbreviations

DHM: Donor human milk
HMB: Human milk bank
HoP: Holder pasteurization
HTST: High temperature short time
NICU: Neonatal intensive care unit
MOM: Mother's own milk
Ig: Immunoglobulin
GF: Growth factor
NEC: Necrotizing enterocolitis
SIgA: Secretory immunoglobulin A
TGF-β_2_: Transforming growth factor-beta 2
EGF: Epidermal growth factor.
