# Supplementary material for: Effect of HTST and Holder Pasteurization on the Concentration of Immunoglobulins, Growth Factors, and Hormones in Donor Human Milk
Source: Front Immunol. 2018 Sep 27;9:2222. doi: 10.3389/fimmu.2018.02222 (PMC6170621; doi:10.3389/fimmu.2018.02222)
Supplement: Supplementary file 1 [file Table_1.docx]

Supplementary Material

**Effect of HTST and Holder pasteurization on the concentration of immunoglobulins, growth factors and hormones present in donor human milk**

**Diana Escuder-Vieco*, Irene Espinosa-Martos, Juan M. Rodríguez, Leónides Fernández†, Carmen Rosa Pallás-Alonso†**

*** Correspondence:** Diana Escuder-Vieco, [diana.e.vieco@gmail.com](mailto:diana.e.vieco@gmail.com)

# Supplementary Tables

**Supplementary Table 1. Effects and interactions of temperature (70, 72, and 75ºC) and time (5, 10, 15, 20, and 25 seconds) of HTST treatments on the retention values of immunoglobulins, growth factors and hormones in HTST-treated DHM as determined by two-way ANOVA tests**

|  | **TEMPERATURE** | | **TIME** | | **TEMPERATURE × TIME** | |
| --- | --- | --- | --- | --- | --- | --- |
|  | ***F-value*** | ***p*** | ***F. value*** | ***p*** | ***F. value*** | ***p*** |
| **IgA** | 2.87 | 0.063 | 1.62 | 0.179 | 0.38 | 0.929 |
| **IgG** | 0.05 | 0.954 | 0.38 | 0.823 | 0.19 | 0.992 |
| **IgM** | 65.46 | 0.000 | 7.53 | 0.000 | 0.15 | 0.996 |
| **EGF** |  |  | 0.16 | 0.955 |  |  |
| **TGF-β_2_** | 4.00 | 0.022 | 0.33 | 0.854 | 0.16 | 0.995 |
| **Adiponectin** | 2.01 | 0.139 | 0.72 | 0.579 | 0.81 | 0.598 |
| **Ghrelin** | 3.90 | 0.026 | 0.57 | 0.685 | 0.27 | 0.973 |
| **Leptin** | 0.96 | 0.386 | 0.16 | 0.957 | 0.20 | 0.989 |
